# Prediction of Advisability of Returning Home Using the Home Care Score

**DOI:** 10.1155/2015/501042

**Published:** 2015-09-29

**Authors:** Akiyoshi Matsugi, Keisuke Tani, Yoshiki Tamaru, Nami Yoshioka, Akira Yamashita, Nobuhiko Mori, Kosuke Oku, Masashi Ikeda, Kiyoshi Nagano

**Affiliations:** ^1^Faculty of Rehabilitation, Shijonawate Gakuen University, Hojo 5-11-10, Daitou City, Osaka 574-0011, Japan; ^2^Department of Rehabilitation, Pegasus Rehabilitation Hospital, Hamaderafunaochohigashi 4-269, Nishi-ku, Sakai-shi, Osaka 592-8341, Japan; ^3^Bobath Memorial Hospital, Department of Rehabilitation, 1-6-5 Higashinakahama, Joto-ku, Osaka 536-0023, Japan; ^4^Department of Rehabilitation, Yamamoto Hospital, Toge 6-7-26, Hashimoto City, Wakayama 648-0072, Japan; ^5^Department of Rehabilitation, Hanna Chuo Hospital, Tawaraguchi 741, Ikoma City, Nara 630-0243, Japan; ^6^Okamoto Home Nursing Station, Ikeda-Asahi-Cho 103-22-24, Neyagawa City, Osaka 572-0035, Japan

## Abstract

*Purpose*. The aim of this study was to assess whether the home care score (HCS), which was developed by the Ministry of Health and Welfare in Japan in 1992, is useful for the prediction of advisability of home care. *Methods*. Subjects living at home and in assisted-living facilities were analyzed. Binominal logistic regression analyses, using age, sex, the functional independence measure score, and the HCS, along with receiver operating characteristic curve analyses, were conducted. *Findings/Conclusions*. Only HCS was selected for the regression equation. Receiver operating characteristic curve analysis revealed that the area under the curve (0.9), sensitivity (0.82), specificity (0.83), and positive predictive value (0.84) for HCS were higher than those for the functional independence measure, indicating that the HCS is a powerful predictor for advisability of home care. *Clinical Relevance*. Comprehensive measurements of the condition of provided care and the activities of daily living of the subjects, which are included in the HCS, are required for the prediction of advisability of home care.

## 1. Introduction

The functional independence measure (FIM) is frequently used to predict the ability of activities of daily living (ADL) after rehabilitation [1–6] and the advisability of returning home after hospitalization [7–9]. A change in the total FIM score of one point is equivalent to approximately 2 minutes of help from another person per day [10], and the total FIM score hence reflects the quantity of necessary care for the patient. In other words, the FIM can generally be used to determine whether a person who has suffered an impediment can return home or has to go to an assisted-living facility after hospitalization [11]. On the other hand, in some cases, even though the FIM score is low, the person who suffered an impediment can still return home if there is adequate support by coresident household members [7, 8]. Thus, whether the patients can return to their own home is based not only on the ability of ADL but also on the available support provided by the coresident household members.

To comprehensively measure the care support conditions and the abilities of ADL, the home care score (HCS) was developed by the study group of the Ministry of Health and Welfare in Japan in 1992 (see Appendix). The factors determined to influence the advisability of home care were extracted based on previous studies associated with home care in Japanese elderly subjects. In other words, the HCS is based on the care provider's condition, including (1) the care provider's health; (2) the availability of a care provider; (3) the availability of a substitute care provider; (4) the care provider's motivation; family/home environment, which includes (5) the bedroom availability, (6) rental house or owned home, (7) the family income, and expected public pension; (8) the patient's general condition, including the abilities to (A) feed oneself, (B) bathe, (C) transfer, (D) dress, and (E) use the toilet; and their (F) verbal communication skills, (G) mental status, (H) medical condition, and (I) motivation (Appendix).

Miyamori and Okajima reported that the HCS at the time of hospital discharge could predict the advisability of home care in Japan [12]. However, it is still not clear whether the precision of the prediction of advisability of home care using the HCS is higher than that using other predictors such as age [13–15], sex [16], number of coresident household members [9, 17], and the FIM [7–9]. Therefore, the first aim of this study was to calculate a high-precision logistic regression equation for the prediction of advisability of home care using various predictive factors, including the HCS. In addition, we investigated whether the precision of the prediction with only the HCS was higher than that with only the FIM, in order to determine which predictor of returning home is the most powerful [7–9], using receiver operating characteristic (ROC) analyses.

Additionally, the circumstances of the care system in Japan have changed greatly after long-term care insurance was introduced in 2000, with the patient need for care having increased [18] along with the available care service [19]. Thus, hospitalized patients can now return home with comprehensive care services provided from suppliers other than their family, even if the ADL of the patient is limited. On the contrary, the family sizes have become smaller and the frequency of elderly care by other elderly persons has increased [20], indicating that the amount of care service provided by the family has decreased. Hence, it is possible that the optimal cut-off point of the HCS for home care has changed from the previously reported cut-off of 11 [12]. With this in mind, the second aim of this study was to investigate the optimal cut-off point of HCS for determining the advisability of home care.

## 2. Methods

The study was performed in April 2014 in Osaka, Japan. The inclusion criteria were as follows: (1) subjects who had been discharged from the hospital more than 6 months prior to the study, (2) subjects who had received certification for long-term care, and (3) subjects who provided informed consent to participate in this study among all patients whom the authors were associated with in April 2014. The exclusion criteria were (1) subjects who had lived at home with care for less than 6 months after hospital discharge and (2) subjects who had missing clinicodemographic data, including age, sex, underlying disease, number of coresidents before care was needed, the FIM score, and the HCS. A total of 148 elderly people met all the above criteria and were included in our analysis.

To test the validity of the HCS and FIM for advisability of home care, we used the known-groups method, with the subjects distributed into two groups based on the characteristics of their residence, that is, home versus facility. The included numbers of subjects who lived at home with care provided by their family and who lived in an assisted-living facility were 76 (38 males and 38 females) and 72 (36 males and 36 females), respectively. The former subjects were defined as the home care group and the latter as the facility care group.

Clinicodemographic parameters, including age, sex, underlying disease, and number of coresidents before care was needed, were assessed retrospectively. The FIM score and HCS were measured after obtaining the participants' consent. All data were collected by the physical therapists or occupational therapists in charge of the subject.

The ADL abilities of each subject were evaluated using the FIM. The HCS was acquired to measure the ability of the family to take care of the subject at home. The factors evaluated in order to obtain the HCS included the availability of a care provider; the care provider's health and motivation; the availability of a substitute care provider; the family income, bedroom availability, and home environment; the patient's general conditions, including the ability to feed oneself and bathe; and the patient's transfer status, verbal communication skills, mental status, medical condition, and motivation (Appendix).

*t*-test was conducted to assess the differences in the mean ages between the home care and facility care groups, after normal distribution was demonstrated using the one-sample Kolmogorov-Smirnov test. The Mann-Whitney test was conducted to assess the differences in the median FIM score and HCS between the groups. An alpha level of 0.05 was used for the statistical analyses.

Further, stepwise binomial logistic regression analysis was conducted to determine whether the advisability of family-based home care could be predicted based on the subjects' age, sex, FIM score, and HCS; again, the alpha level was set at 0.05 for the statistical analyses. To determine the optimal cut-off scores of the FIM and HCS, ROC analyses were conducted. The sensitivity and specificity were determined for each possible cut-off point. In addition, the area under the curve (AUC) was calculated for each ROC curve. The optimal cut-off points were obtained from the Youden index [maximum (sensitivity + specificity − 1)] and the point on the ROC curve closest to (0, 1), which was calculated as the minimum value of the square root of [(1 − sensitivity) (1 − sensitivity)] + [(1 − specificity) (1 − specificity)]. A greater accuracy is reflected by a larger Youden index and a smaller distance to (0, 1) [21]. Lastly, DeLong's test for two correlated ROC curves was conducted to determine whether there was any difference between the AUCs of the HCS and FIM. All statistical analyses were performed with R (version 2.13.0; the R Foundation for Statistical Computing, Vienna, Austria).

The ethics committee of Shijonawate Gakuen University approved all study protocols, and the study was conducted in accordance with the declaration of Helsinki. The patient information was entirely coded in order to ensure the anonymity of the subjects.

## 3. Results

**Figure 1:**
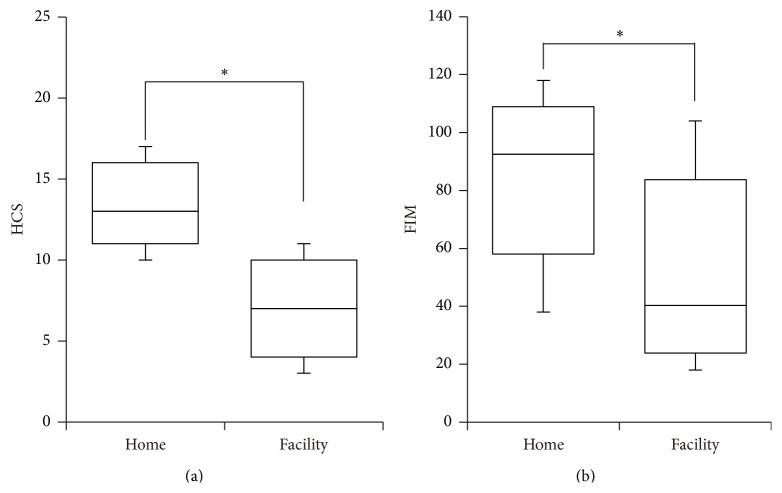
Boxplots of the home care score (HCS) (a) and functional independence measure (FIM) score (b). The middle horizontal lines indicate the median; the top and bottom lines of the box indicate the tertiary and first quartiles, respectively; and the top and bottom vertical lines indicate 90% and 10%, respectively. Asterisks indicate significant differences.

Table 1 shows the characteristics of the subjects in the home care and facility care groups. The mean ages (±standard deviation) of the patients in the home care and facility care groups were 74.2 ± 10.8 and 77.7 ± 10.6 years, respectively. There was no significant difference in age between the groups (*t* = −2.0, d.f. = 146, *p* = 0.0501). The median (±quartile deviation) FIM scores in the home care and facility care groups were 92.5 ± 58 and 40.5 ± 23.75, respectively. The Mann-Whitney test revealed that the FIM score in the home care group was significantly higher than that in the facility care group (*Z* = 5.026, *p* < 0.001). The median HCS in the home care and facility care groups were 13 ± 11 and 7 ± 4, respectively, and the Mann-Whitney test revealed that the HCS in the home care group was significantly higher than that in the facility care group (*Z* = 8.35, *p* < 0.001) (Figure 1).

Using stepwise binominal logistic regression analysis, the only significant parameter was found to be the HCS, which was subsequently included in the logistic regression equation (*p* < 0.001, odds ratio = 1.64, 95% confidence interval = 1.40–1.92). The correlation coefficient was determined as 0.70, and the regression coefficient was 0.49 (likelihood ratio = 84.7, d.f. = 1, *p* < 0.001).

**Figure 2:**
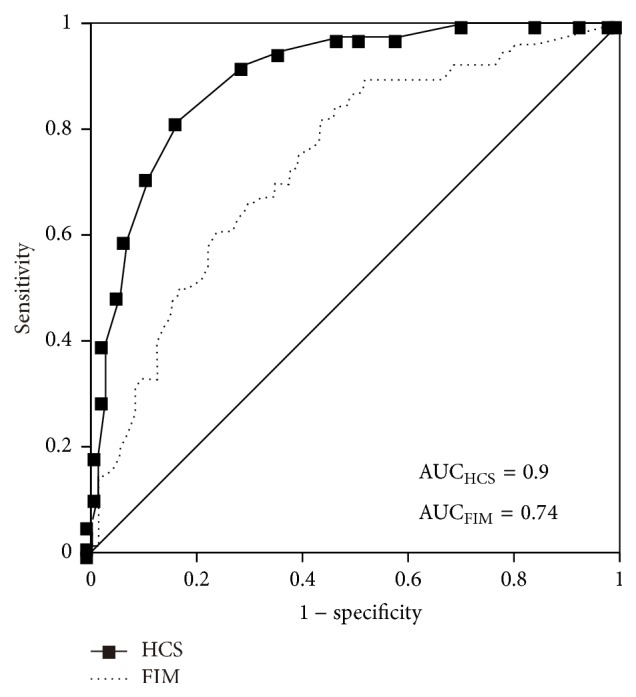
Operating characteristic curves of the home care score (HCS) and functional independence measure (FIM). AUC, area under the curve.

The results of the ROC curve analyses of the different cut-off points of the HCS score for identifying subjects, including the Youden index, distance of the ROC curve to point (0, 1), sensitivity, specificity, and positive predictive value, are shown in Table 2. The ROC curves in Figure 2 were constructed based on the data of the HCS and FIM score. The AUCs for the HCS and FIM score were 0.9 and 0.74, respectively. DeLong's test revealed that the AUC of the HCS was significantly larger than the AUC of the FIM. The optimal cut-off points of the HCS and FIM were 11 and 48, respectively. These points were selected based on the maximum Youden indices for the HCS and FIM, which were 0.65 (sensitivity = 0.82, specificity = 0.83) and 0.39 (sensitivity = 0.82, specificity = 0.57), respectively. Using these optimal cut-off points, the positive predictive values of the HCS and FIM were found to be 0.84 and 0.67, respectively.

## 4. Discussion

The binominal logistic regression equation for the prediction of advisability of home care, which was calculated using age, sex, the FIM score, and the HCS, resulted in only the HCS being significant and selected as a parameter. Furthermore, the AUC of ROC for the HCS was significantly larger than that for the FIM score, indicating that the precision of prediction with the HCS was higher than that with the FIM score, which has also been previously demonstrated to be a strong predictor of the advisability of home care [7–9]. If the AUC is between 0.7 and 0.9, the performance to distinguish is considered moderate, whereas if the AUC is greater than 0.9, the performance to distinguish is considered high [22]. In this study, the AUC of ROC of the FIM was 0.74, while that of the HCS was 0.9. Based on these findings, the precision of prediction with the FIM can be considered moderate. On the other hand, the precision of prediction with the HCS can be considered high, suggesting that the HCS is a more powerful predictor for advisability of returning home from the hospital.

Smith et al. previously reported that patients with low FIM scores could return home if adequate care could be provided by the coresidential family [11], indicating that the FIM, which cannot evaluate the quantity of care provided by the family, may be insufficient for the prediction of advisability of home care. On the other hand, the HCS can not only measure the ADL of the subject but also comprehensively measure the conditions of the provided care. Therefore, our results suggest that a scale that can comprehensively evaluate the ADL and the condition of the provided care, such as the HCS, is necessary to successfully predict the advisability of home care.

It should be noted that the HCS was established in Japan, and the factors that constitute the HCS were extracted based on previous studies on home care for Japanese elderly. For example, the factor on family income was extracted based on an important study on the association between family income and home care for elderly people by the welfare development center for the elderly in Japan in 1985. The income item is scored as 1 when there is any family income, except public pension, because it was described in the above-mentioned report that the presence of family income except public pension is advantageous to home care. In other words, the HCS was established in accordance with the circumstances of the Japanese care system. Therefore, the scale may need to be revised in accordance with the circumstances and culture of each country.

Moreover, all participants in this study were Japanese people living in Osaka, and long-term care insurance is currently available in Japan. Hence, it is necessary to conduct similar studies in countries other than Japan to confirm the generalizability of our results. In particular, the population is aging all over the world, and poor ability of providing home care may prevent return to home from the hospital in other countries as well, in the same way as in Japan. Therefore, the ability of providing care should be evaluated, for example, by using the HCS, in order to create a plan for the patients to return home from the hospital in these different countries.

In conclusion, comprehensively measuring the condition of the provided care and the ADL ability of the subjects, for example, using the HCS, is required for accurate prediction of advisability of home care. Based on our results, we conclude that the HCS is essential to create appropriate rehabilitation plans. Furthermore, in order to enable elderly or disabled subjects to live in their own home with care, attempts to raise the HCS should be made.

## Figures and Tables

**Table 1:** Characteristics of the subjects.

	Home	Facility
Number of subjects		
Male	38	36
Female	38	36
Age (years)	74.2 ± 10.8	77.7 ± 10.6
Disease/condition		
Cerebrovascular disease	48	40
Osteoarticular disease	16	7
Intractable disease	5	3
Respiratory disease	0	6
Senility	0	5
Metabolic disease	1	3
Dementia	2	2
Heart disease	1	2
Spinal cord injury	3	0
Carcinoma	0	2
Gastrointestinal disease	0	2
FIM score	92.5 ± 58	40.5 ± 23.8
HCS	13 ± 11	7 ± 4

FIM, functional independence measure; HCS, home care score.

**Table 2:** Receiver operating characteristic (ROC) curve analysis of the home care score (HCS) to determine the most appropriate cut-off score.

Cut-off	Home	Facility	Total	Youden index	Distance to ROC curve	Sensitivity	Specificity	Positive predictive value
1	0	0	0	0.00	1.00	1.00	0.00	0.51
2	0	1	1	0.01	0.99	1.00	0.01	0.52
3	0	4	4	0.07	0.93	1.00	0.07	0.53
4	0	6	6	0.15	0.85	1.00	0.15	0.55
5	0	10	10	0.29	0.71	1.00	0.29	0.60
6	2	9	11	0.39	0.58	0.97	0.42	0.64
7	0	5	5	0.46	0.51	0.97	0.49	0.67
8	0	3	3	0.50	0.47	0.97	0.53	0.69
9	2	8	10	0.59	0.36	0.95	0.64	0.73
10	2	5	7	0.63	0.30	0.92	0.71	0.77
11	8	9	17	0.65	0.25	0.82	0.83	0.84
12	8	4	12	0.60	0.31	0.71	0.89	0.87
13	9	3	12	0.52	0.41	0.59	0.93	0.90
14	8	1	9	0.43	0.52	0.49	0.94	0.90
15	7	2	9	0.37	0.61	0.39	0.97	0.94
16	8	0	8	0.26	0.71	0.29	0.97	0.92
17	8	1	9	0.17	0.82	0.18	0.99	0.93
18	6	0	6	0.09	0.89	0.11	0.99	0.89
19	4	1	5	0.05	0.95	0.05	1.00	1.00
20	3	0	3	0.01	0.99	0.01	1.00	1.00
21	1	0	1	0.00	1.00	0.00	1.00	—

**Table 3:** Components of the home care score.

(1) Care provider's health	Sickly (0) Healthy (1)
(2) Availability of a care provider	Not available (0) Available (1)
(3) Availability of a substitute care provider	Not available (0) Available (1)
(4) Care provider's motivation	Poor (0) Normal (2) Good (4)
(5) Bedroom availability	Not available (0) Available (1)
(6) Home environment	Rental house (0) Owned house (1)
(7) Family income, except public pension	Nonexisting (0) Existing (1)
(8) Patient's general condition	
(A) Ability to feed oneself	Dependent (0) Independent (1)
(B) Bathing	Dependent (0) Independent (1)
(C) Transfer	Dependent (0) Independent (1)
(D) Dressing	Dependent (0) Independent (1)
(E) Toilet use	Dependent (0) Independent (1)
(F) Verbal communication skills	Poor (0) Good (1)
(G) Mental status	Poor (0) Good (2)
(H) Medical condition	Poor (0) Good (1)
(I) Patient's motivation	Poor (0) Normal (1) Good (2)

## Appendix

See Table 3.
